# Two Highly Conserved Cysteine Residues in HPV16 L2 Form an Intramolecular Disulfide Bond and Are Critical for Infectivity in Human Keratinocytes

**DOI:** 10.1371/journal.pone.0004463

**Published:** 2009-02-13

**Authors:** Samuel K. Campos, Michelle A. Ozbun

**Affiliations:** The Department of Molecular Genetics and Microbiology, The University of New Mexico School of Medicine, Albuquerque, New Mexico, United States of America; The Rockefeller University, United States of America

## Abstract

**Background:**

Minor capsid protein L2 performs an indispensable but uncharacterized role in human papillomavirus infections. A neutralizing B cell epitope has recently been mapped to the N-terminus of HPV16 L2, residues 17–36, and exposure of this region of L2 has been implicated in translocation of incoming virions from the endo/lysosomal compartment to the cellular cytoplasm. Here we examine the redox state of Cys22 and Cys28 two highly conserved cysteines located within this epitope. We also investigate the infectivity of virions containing L2 single and double cysteine point mutants.

**Methodology and Principal Findings:**

Denaturing/non-reducing gel analysis and thiol labeling experiments of wild type and cysteine mutant HPV16 virion particles strongly support the existence of a buried intramolecular C22–C28 disulfide bond. The disulfide was confirmed by tandem mass spectrometry of L2 protein from non-reduced virions. Single C22S and C28S and the double C22/28S mutants were non-infectious but had no apparent defects in cell binding, endocytosis, or trafficking to lysosomes by 8 h post infection. During infection with L2 mutant particles, there was a marked decrease in L2 levels compared to wild type L2-containing virions, suggesting a failure of mutant L2/genome complexes to exit the endo/lysosomal compartment.

**Conclusions and Significance:**

L2 residues C22 and C28 are bound as an intramolecular disulfide bond in HPV16 virions and are necessary for infectivity. Previous work has suggested that the furin-dependent exposure of the 17–36 epitope and subsequent interaction of this region with an unknown receptor is necessary for egress from the endo/lysosomal compartment and infection. Identification of the C22–C28 disulfide suggests that reduction of this disufide bond may be necessary for exposure of 17–36 and HPV16 infection.

## Introduction

Human papillomaviruses (HPVs) are small non-enveloped DNA viruses and are the causative agents of cervical cancer, with HPV type 16 being responsible for more than 50% of cervical cancers in women worldwide [1], [2]. The HPV16 virion consists of a ∼8 kb circular dsDNA genome packaged into a ∼60 nm icosahedral capsid. Initial attachment of HPV16 virions to the host cell membrane appears to occur via heparan-sulfate proteoglycans (HSPGs) [3], [4]. The capsid is then thought to undergo conformational changes resulting in transfer to an unknown non-HSPG entry receptor [5], [6]. Additional studies have also implicated direct binding to the extracellular matrix (ECM) and transfer to the cell membrane via laminin-5 [7], a process which may also involve activation of and transfer along cell surface filopodia [8], [9]. Cellular endocytosis of bound HPV16 virions is thought to occur via a classical clathrin-mediated pathway with trafficking through the endosomal/lysosomal compartments [10], [11]. A recent report observed HPV16 entry through a clathrin/caveolin-independent mechanism involving tetraspanin-enriched microdomains, although this study was done with HeLa cervical carcinoma, 293TT embryonic kidney, and HuH7 liver cell lines [12]. Regardless of the mode of entry, trafficking through the acidic endo/lysosomal pathway appears to be critical for infection of multiple papillomavirus (PV) types and the decrease in pH is likely a trigger for virion uncoating and exit from this compartment [10], [13], [14].

The capsid is comprised of 72 pentamers of the major capsid protein L1 and up to 72 molecules of the minor capsid protein L2, localized along the inner surface of the capsid, within the central cavities beneath the L1 pentamers [15], [16]. Biochemical and structural studies show that mature virions exist in an oxidized state with adjacent L1 pentamers crosslinked together by disulfide bonds, stabilizing the capsid structure [16]–[20]. The L2 protein is critical for HPV16 infection [21] and is a multifunctional protein, having roles in genome encapsidation [22], [23], L1 interaction and capsid stabilization [24], [25], and endosomal escape of virions [13]. L2 protein from incoming particles has been shown to colocalize with viral genomes and nuclear promyelocytic leukemia protein (PML) bodies [26], and L2 may function to escort the viral genome into the host cell nucleus, perhaps through direct interactions with various nuclear import receptors and the cellular chaperone Hsc70 [27], [28]. L2-dependent localization of viral genome to PML bodies may serve to promote efficient early gene expression for the establishment of infection. L2 may also play a role in late gene expression during HPV infection by directly binding the viral E2 protein and repressing its transcriptional activation functions [29], [30]. L2-dependent recruitment of E2 and viral genomes to PML bodies is also postulated to function in assembly of progeny virions during the late phases of HPV infection, through sequestration of virion components [31]. L2 has also been implicated in various interactions with host factors including cell surface binding [32], [33], actin and dynein binding [34], [35] syntaxin 18 binding [36], and cleavage by the cellular protease furin [37].

The HPV16 L2 protein contains only two cysteines, C22 and C28. Both are absolutely conserved across all known mammalian and avian PV types. Recently, L2 residues 17–36 have been identified as a B-cell epitope that is capable of eliciting a cross-neutralizing antibody response against a broad variety of HPV types, making the antigen a promising vaccine candidate [38], [39]. Given the position of C22 and C28 within this neutralizing antibody epitope and the highly oxidized state of mature particles we sought to examine the redox state of C22 and C28 in HPV16 virions and their role in infectivity. We found that C22 and C28 exist as a buried intramolecular disulfide in mature virions. C22S and C28S single and C22/28S double mutant virions were non-infectious but exhibited normal cell binding, uptake, and lysosomal colocalization at 8 h post infection. Interestingly, the mutant L2 proteins were degraded more rapidly than wild type L2 during the course of infection. We hypothesize that the cysteine mutants may be defective for endosomal escape, resulting in lysosomal degradation of L2/genome complexes wherein infection is blocked.

## Results

### Conserved L2 cysteines are not accessible for BMCC-biotin modification

**Figure 1:**
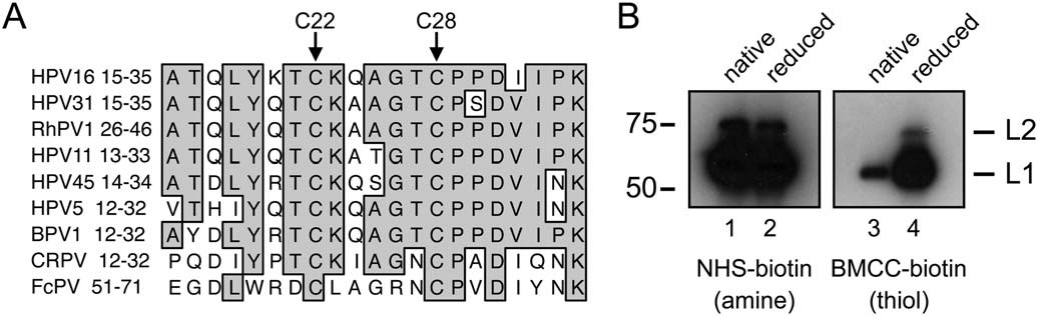
Highly conserved HPV16 L2 cysteines C22 and C28 are not reactive with BMCC-biotin in native virions. (A) Primary amino acid sequence alignment of L2 proteins from various members of the Family *Papillomaviridae* shows conservation of C22 and C28 (HPV16) across divergent *genera*. (B) Amine and thiol biotinylation assays of HPV16 virions. Purified virions were either mock treated (native) or DTT treated (reduced) prior to incubation with amine reactive NHS-biotin or thiol reactive BMCC-biotin. Biotinylated proteins were separated by SDS-PAGE, transferred to membranes, and detected with streptavidin-HRP. Note that L2 cysteines are only reactive when the virions are reduced prior to labeling.

Sequence alignment of diverse papillomavirus types shows that C22 and C28 of HPV16 L2 are absolutely conserved throughout the Family *Papillomaviridae* (Fig. 1A), prompting us to further examine the role of these two cysteines in HPV infection. To examine the native redox state of the two highly conserved L2 cysteines within the structural context of virions, we performed a thiol-specific biotinylation assay. Purified HPV16 virions were kept in their native state or reduced by DTT prior to chemical biotinylation with either amine-specific NHS-biotin or thiol-specific BMCC-biotin reagents. Biotinylated proteins were then detected by western blot and streptavidin-HRP staining. NHS-biotin labeling resulted in robust biotinylation of the amine groups of both L1 and L2 capsid proteins, irrespective of the redox status of the virions (Fig. 1B, lanes 1–2). In contrast, L2 was only labeled detectably with BMCC-biotin if the virions were first reduced by DTT (Fig. 1B, lane 4). This suggests that within the context of intact particles, C22 and C28 of HPV16 L2 are either buried and inaccessible to BMCC-biotin or they are bound up as disulfides, S-nitrosothiols (S-NO), or some other S-alkyl modification. These possibilities are not mutually exclusive, as the cysteines could be both modified and buried.

### Characterization of L2 cysteine mutant virions

**Figure 2:**
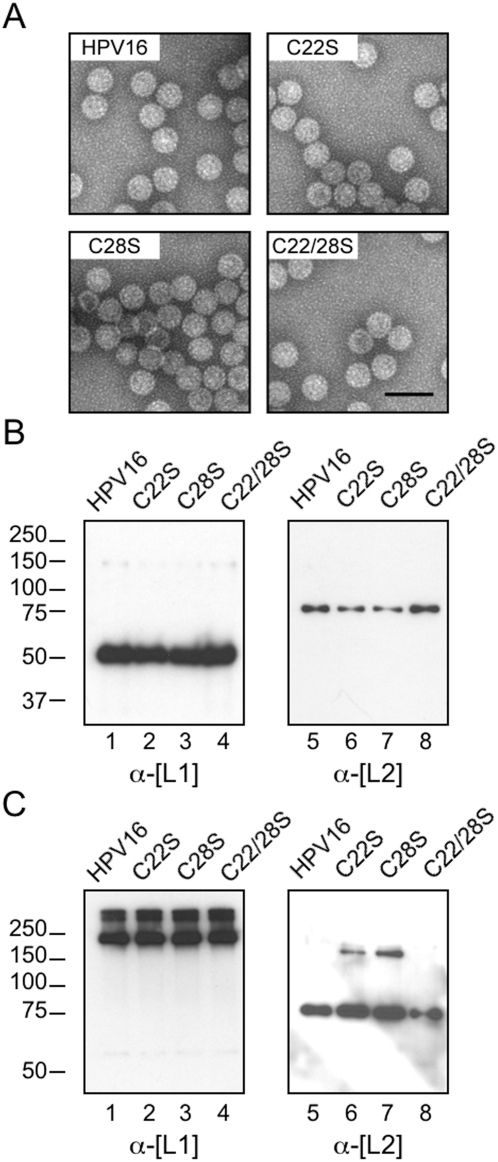
Characterization of L2 cysteine mutant virions. (A) Morphology of the L2 wild type and cysteine mutant virions. CsCl-purified virion preparations were negatively stained and visualized by transmission electron microscopy. Scale bar represents 100 nm. (B) Reducing and denaturing SDS-PAGE of wild type and mutant L2-containing virions, immunoblotted for L1 (left panel) and L2 (right panel) capsid proteins. Note the absence of L2 size shift for any of the cysteine mutants, suggesting no large cysteine modifications are present. (C) Analysis of HPV16 L2 wild type and mutant virions by denaturing SDS-PAGE under non-reducing conditions. L1 immunoblot (left panel) shows disulfide-linked multimers of L1 for wild type and mutant virions. L2 immunoblot (right panel) shows no detectable L2-L2 dimers for wild type or C22/28S virions (lanes 1,4), but a fraction of L2 in the single cysteine mutants exist as disulfide-linked L2-L2 dimers (lanes 2,3).

Single C22S and C28S and the double C22/28S cysteine-to-serine point mutations were generated in the L2 gene of the HPV16 capsid expression plasmid. Mutant virions were produced by transfection of 293TT cells [40], [41] and purified by CsCl density gradient centrifugation as previously described [11]. Virions containing mutant L2 proteins were obtained in yields comparable to normal HPV16 virions containing wild type L2. SDS-PAGE and Coomassie staining-based densitometry indicated that each of the virus preparations contained an average of ∼22 molecules L2 per virion (data not shown). Visualization by transmission electron microscopy revealed normal capsid morphology among all L2 mutant virions (Fig. 2A) and dot blot analysis showed no decrease in genome encapsidation for any of the mutants (data not shown). SDS-PAGE and immunoblotting of virion preparations for L1 and L2 revealed no defects in L2 encapsidation (Fig. 2B, lanes 5–8). The absence of any size shift in the L2 mutants suggested that no large modifications (palmitoylation, prenylation, etc.) were present on the wild type L2 cysteines.

Several possibilities exist regarding the nature of potential disulfide linkages involving C22 and C28 in the L2 protein. The cysteines could be linked as intermolecular disulfides to form L2-L2 dimers or they could be bound as an intramolecular disulfide to form a hairpin-like structure. L2 could also form a disulfide link to L1, which contains twelve cysteine residues. Alternatively, the L2 cysteines could be bonded to small molecules like glutathione or free cysteine as mixed disulfides or bound to nitric oxide (NO) as an S-NO group. To address these possibilities, denaturing SDS-PAGE was performed under non-reducing conditions to visualize any disulfide-linked proteins in the virions containing wild type and mutant L2 proteins (Fig. 2C). L1 was present as high molecular weight multimers in all the virion preparations (Fig. 2C, lanes 1–4). L2 was present only as monomers in wild type virions, excluding the possibilities of disulfide linked L2-L2 dimers or L2-L1 complexes (Fig. 2C, lane 5). As expected the double mutant was only detected as a monomer (Fig. 2C, lane 8). Interestingly, the single C22S and C28S mutants were present mainly as L2 monomers although a small fraction existed as disulfide linked 150 kDa L2-L2 dimers (Fig. 2C, lanes 6–7). Recent cryoEM data from Buck *et al.* suggest a maximum stoichiometry of one L2 molecule per L1 pentamer or 72 L2 molecules per virion [15]. L2 is therefore present at ∼30% occupancy in each of our preparations, making it likely that the L2-L2 dimers may represent the small fraction of the total L2 molecules that lie close enough in space within the 72 L1-pentamer lattice to form intermolecular disulfide linkages. The absence of intermolecular L2 dimers in wild type HPV16 virions suggests that C22 and C28 are bound either as an intramolecular disulfide hairpin or as conjugates to small molecules, molecular forms that could potentially have the same migration pattern as reduced L2 in a polyacrylamide gel [42]. However, the L2-L2 dimerization in the C22S and C28S mutant virions argues that the cysteines are normally present as an intramolecular disulfide; if they were bound as small molecule mixed disulfides or S-NO groups, then the single mutants would not be capable of L2-L2 dimerization because the single remaining cysteine would still be bound up and unavailable for disulfide linkage.

### L2 cysteines are present as an intramolecular disulfide hairpin

**Figure 3:**
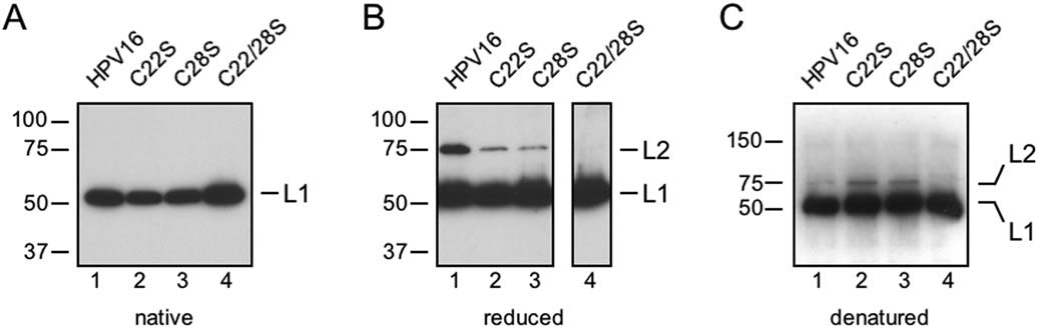
Thiol biotinylation assay of HPV16 L2 cysteine mutants. (A) Lack of L2 biotinylation by BMCC-biotin of normal and mutant virions in their native state suggest that L2 cysteines are either buried and inaccessible to BMCC-biotin or bound up and not present as free thiols. (B) L2 cysteines become accessible and are biotinylated in DTT-reduced virions. (C) Denaturation of virions by SDS results in L2 biotinylation of C22S and C28S single mutants only (lanes 2,3), indicative of free but buried cysteines in these mutants. Lack of L2 biotinylation in denatured wild type virions implies that although the cysteines are exposed, they are bound as an intramolecular disulfide, becoming available for modification in the single mutants only.

Thiol-specific biotinylation experiments were performed to further elucidate the nature of HPV16 L2 C22 and C28 residues. Virions were kept in their native state, reduced by DTT, or denatured with SDS prior to BMCC-biotin labeling. As seen previously, L2 molecules from virions in their native state were not labeled by BMCC-biotin (Fig. 3A) and reduction of virions was required for L2 biotinylation (Fig. 3B). For the reduced samples, L2 biotinylation was less pronounced for the C22S and C28S mutants than for virions containing wild type HPV16 L2 proteins (Fig. 3B, lanes 2–3), presumably because single mutants have only half the number of cysteines available for BMCC-biotin modification. As expected, no labeling was observed for the double C22/28S mutant, which lacks cysteine residues (Fig. 3B, lane 4). If C22 and C28 were normally bound together as an intramolecular disulfide one might predict that the lone cysteines of the single C22S and C28S mutants in their native virion states would be free and available to react with BMCC-biotin. Yet, biotinylation was only observed upon virion reduction with DTT (Fig. 3A & B, lanes 2–3). Inaccessibility of the L2 cysteines in native virion paticles could explain this lack of BMCC-biotin labeling. To determine if C22 and C28 are simply buried in the context of native virions, virions were denatured with SDS treatment prior to BMCC-biotin labeling. Only the C22S and C28S single mutants exhibited robust L2 biotinylation upon denaturation with SDS, indicating that a majority of the lone cysteines of the C22S and C28S single mutants exist as free thiols (Fig. 3C, lanes 2–3). L2 proteins of wild type HPV16 virions were only minimally labeled after denaturation, suggesting that although C22 and C28 are accessible, they are disulfide bonded (Fig. 3C, lane 1). Again no labeling was seen for the C22/28S double mutant because no cysteines are present (Fig. 3C, lane 4).

**Figure 4:**
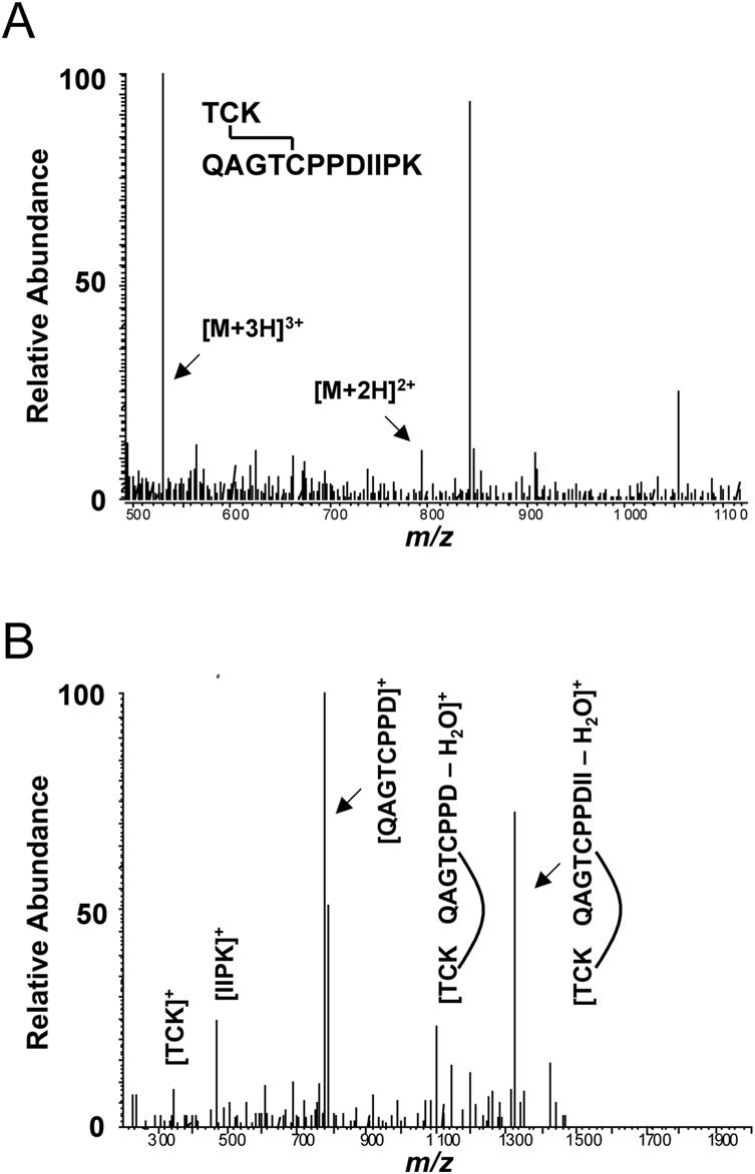
Mass spectrometry of non-reduced L2 protein. (A) The full MS spectra of the L2 tryptic peptides T21-K23 and Q24-K35 with C22–C28 disulfide bond intact. The predominant doubly charged and triply charged parent cations are evident. (B) The MS/MS spectra of the doubly charged precursor ion and assignment of internal fragments. Upon collisional activation the disulfide bond was broken, but two major non-reduced species are evident. The non-reduced species were preferentially fragmented at I32 and P34.

Tandem mass spectrometry (MS/MS) of non-reduced L2 sample was performed to verify the existence of the C22–C28 disulfide. Purified wild type HPV16 virions were reacted with excess N-ethylmaleimide (NEM) to protect any native disulfide bonds from reduction by reactive free thiols. Virion proteins were then separated by denaturing, non-reducing SDS-PAGE and the L2 band was excised for in-gel trypsin digestion and extraction of tryptic peptides. Trypsin cleavage of L2 is predicted to occur following lysine residues K20, K23, and K35, within the immediate vicinity of C22 and C28, in the L2 primary amino acid sequence. K23 is positioned between C22 and C28, so the disulfide will link the adjacent T21-K23 and Q24-K35 tryptic peptides. MS analysis detected masses corresponding to both the doubly and triply charged cations of the disulfide-bonded peptides (Fig. 4A). MS/MS of the doubly charged peptide cation and analysis of the internal fragments further verified its assignment, confirming the existence of the C22–C28 disulfide bond in L2 (Fig. 4B).

### Infectivity of HPV16 virions containing L2 cysteine mutants

**Figure 5:**
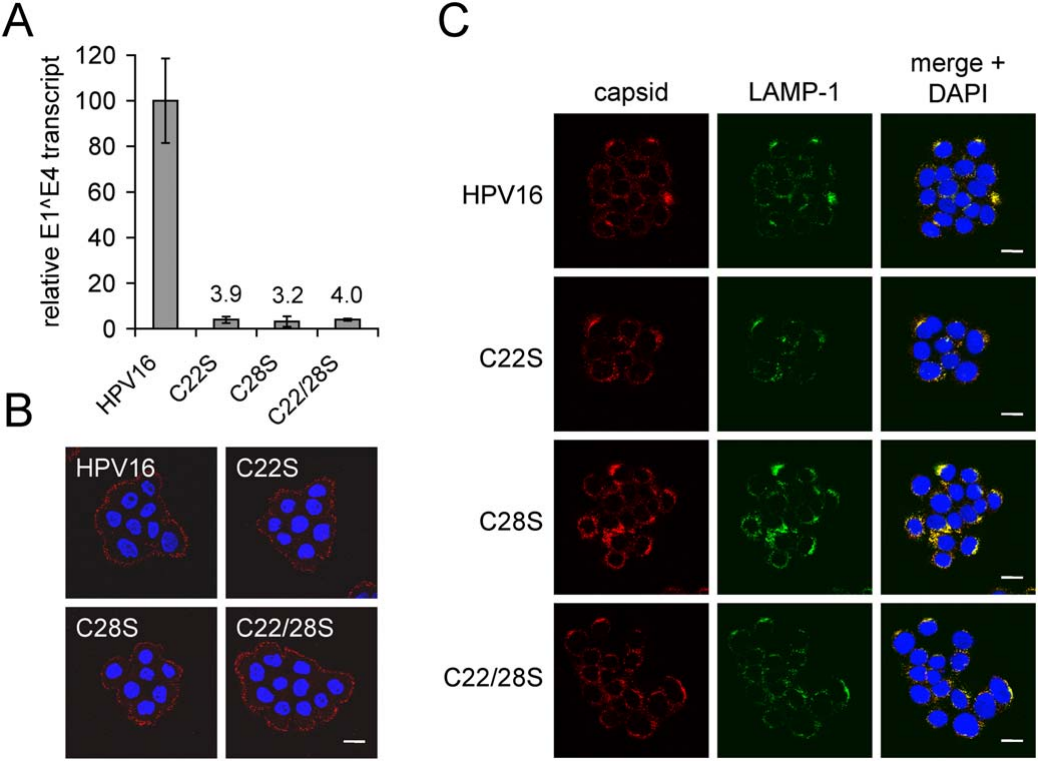
Infectivity, plasma membrane binding, and trafficking are not obviously affected in HPV16 virions containing L2 cysteine mutants. (A) HaCaT cells were exposed to normal and mutant virions at an dose of 100 vge/cell. Total RNA was harvested 48 h post infection and viral E1̅E4 transcripts were quantified by RT-qPCR. Results were normalized relative to wild type HPV16 virion infections and presented as mean values for three replicates±standard deviation. (B) HaCaT cells exposed to AF594-virions (red) for 1 h at 4°C. Unbound virions were washed away prior to fixation. Nuclei were stained with DAPI (blue) and confocal microscopy was performed with the focal point at the plasma membrane. (C) Colocalization of AF594-virions with LAMP-1 after 8 h of trafficking. HaCaT cells bound with AF594-virions (red) were allowed to internalize virus for 8 h at 37°C prior to fixation, permeabilization and immunostaining for LAMP-1 (green). Nuclei were stained with DAPI (blue). Scale bars represent 20 µm.

Papillomavirus virions assembled in the 293TT cell system retain full infectivity and susceptibility to antibody neutralization in experimental infections performed both *in vitro* and *in vivo* [11], [41], [43]. The infectivity of wild type and mutant L2-containing virions encapsidating HPV16 genomes was assessed on subconfluent HaCaT cells, a spontaneously immortalized keratinocyte line commonly used to determine HPV infectivity *in vitro* [11], [44], [45]. Total RNA was harvested from infected HaCaT cells and levels of spliced E1̅E4 viral transcripts were quantified by RT-qPCR, as we have described [11], [14], [46]. Results showed a >95% reduction in infectivity for all L2 cysteine mutants (Fig. 5A). To verify the results were not due to mutant L2 effects on E1̅E4 transcription from the viral genome, this experiment was repeated using wild type and L2 mutant reporter viruses, which encapsidate a luciferase expression plasmid. Results showed a >99.9% decrease in luciferase activity (data not shown) verifying that the L2 cysteine residues are critical for HPV16 virion infectivity.

### Binding and trafficking of cysteine mutants

Purified virions were labeled with amine-reactive AlexaFluor 594 (AF594) and cell binding and uptake were assessed by confocal microscopy. We previously showed that AF594 labeling does not disrupt HPV particle binding to the extracellular matrix (ECM) or cell plasma membrane, antibody-mediated neutralization, internalization time, or infectivity [11]. HaCaT cells were exposed to AF594-virions for 1 h at 4°C to permit binding but not internalization. Mutant and wild type virions all displayed normal ECM and cell membrane binding on HaCaT human keratinocytes (data not shown and Fig. 5B, respectively). Previous work has demonstrated that wild type HPV16 virions enter cells and traffic to perinuclear lysosomal compartments by 8 h post-infection [10], [11]. Wild type and mutant L2-containing AF594-virions were analyzed for cell uptake and trafficking by adding fresh media and shifting the temperature to 37°C for 8 h. Samples were fixed, permeabilized, and immunostained for LAMP-1, a late endosomal/lysosomal marker [47]. None of the mutants displayed any defects in entry or colocalization with LAMP-1 (Fig. 5C), suggesting the infectious block of the cysteine mutants does not involve cell binding or intracellular trafficking.

### Degradation of capsid proteins during intracellular trafficking

**Figure 6:**
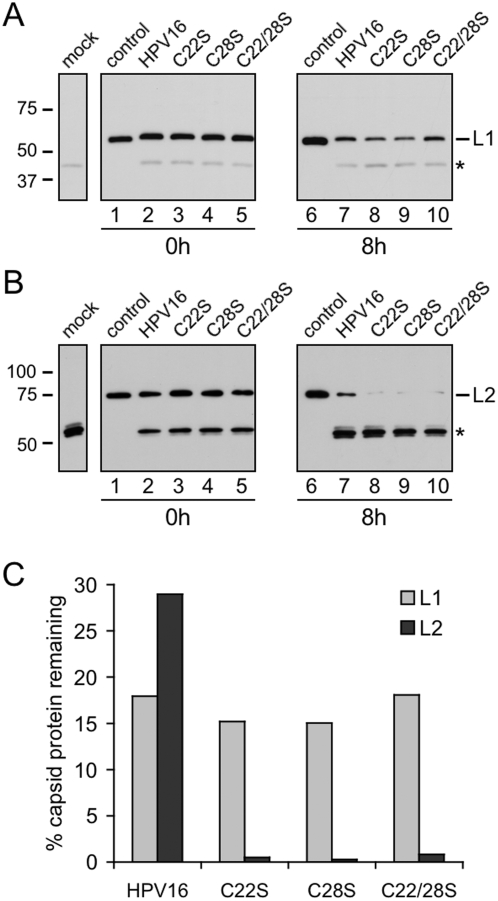
Degradation of wild type and L2 mutant virions during infection. Total cell lysates of infected HaCaT cells were collected at 0 h and 8 h post infection. Lysates were separated by SDS-PAGE and immunoblotted for capsid proteins. (A) L1 immunoblot of mock treated and infected HaCaT cell lysate. (B) L2 immunoblot of mock treated and infected cell lysates. Control lanes contain a known amount of purified HPV16 for comparison across blots. Asterisks represent non-specific bands present in cell lysates. (C) Percentages of L1 (grey bars) and L2 (black bars) capsid proteins remaining after 8 h of intracellular trafficking was calculated by densitometry as described in materials and methods.

AF594 conjugation results primarily in the labeling of the major capsid protein L1 due to its high copy number in the virion and accessible surface location; although L2 and histones are labeled, levels are significantly lower (unpublished observations). The majority of the AF594 signal in microscopy experiments therefore is likely to reflect L1, and this signal may persist even if L1 is proteolytically degraded. To examine the fate of the capsid proteins during cell entry and trafficking, cells were exposed to virus and whole cell lysates harvested at 0 h and 8 h post infection were subjected to immunoblotting for L1 and L2. Known amounts of purified HPV16 virions were resolved on the gels as a control to which the intensities of the sample bands on the same blot could be compared and relative differences in band intensity across different blots determined. Initial particle binding was equivalent among the HPV16 wild type and different L2 mutants (Fig. 6A, lanes 1–5) in agreement with the confocal microscopy data (Fig. 5B). After 8 h of entry and trafficking, L1 levels dropped to similar levels among the HPV16 wild type and mutant particles (Fig. 6A, lanes 6–10), most likely representing degradation of the protein in the lysosomal compartment, where the majority of L1 colocalizes at 8 h (Fig. 5C). Initial levels of L2 were also comparable among the different mutants again demonstrating no defects in L2 levels in the virus particles or in cell binding (Fig. 6B, lanes 1–5). However, during the course of infection, L2 levels of mutant particles were considerably diminished relative to those of wild type L2-containing HPV16 virions (Fig. 6B, compare lane 7 to lanes 8–10). Densitometric comparison of protein levels relative to the blot-internal loading controls enabled relative assessment of samples across blots and the percent of capsid protein remaining after 8 h of intracellular trafficking was determined for normal HPV16 and each of the mutants. Analysis showed that ∼15–20% of the intact L1 protein remained for each of the virus infections (Fig. 6C, grey bars). In contrast, L2 levels for wild type HPV16 virions were ∼25–30% of the input quantity after 8 h of infection, while <1% of intact L2 proteins remained for all the cysteine mutants (Fig. 6C, black bars).

## Discussion

Minor capsid protein L2 plays a critical role in the early events of HPV infection although its precise functions in HPV entry, intracellular trafficking, endosomal escape, and nuclear import of the HPV genome have not been elucidated. Recently a cross-neutralizing B-cell epitope has been mapped to residues 17–36 of HPV16 L2 and RG-1, a monoclonal antibody against this epitope has been characterized [39]. Here we studied two highly conserved cysteine residues, C22 and C28, located within the RG-1 epitope. The redox status of L2 was studied by non-reducing SDS-PAGE and thiol-specific BMCC-biotin labeling experiments of HPV16 wild type and L2 cysteine mutant virions. Taken together, results strongly suggest that C22 and C28 are bonded as a buried intramolecular disulfide in wild type virions. Mutation of either C22 or C28 in L2 results in free cysteines that react with BMCC-biotin when denatured by SDS (Fig. 3C, lanes 2–3). A small percentage of these C22S or C28S mutant L2 molecules can actually form L2-L2 dimers through an intermolecular disulfide (Fig. 2C, lanes 6–7). Wild type L2 can neither form L2-L2 dimers nor react with BMCC-biotin after SDS-denaturation because C22 and C28 are bound together as a disulfide (Fig. 2C, lane5 & 3C, lane 1). Consistent with these conclusions, the C22–C28 intramolecular disulfide was directly observed in non-reduced wild type L2 by tandem MS/MS analysis of polyacrylamide gel purified protein (Fig. 4).

All L2 cysteine mutant virions were non-infectious despite exhibiting normal cell binding, internalization, and trafficking to LAMP-1 positive lysosomes by 8 h post infection (Fig. 5). This finding is not entirely unexpected since previous work suggests that HPV16 virus like particles (VLPs), composed entirely of major capsid protein L1 bind and enter host cells normally, trafficking to the lysosome [10]. During the first 8 h of infection, we detected notable differences in the degradation of incoming wild type and mutant L2 proteins. While L2 levels decreased to ∼30% the input for wild type virions during the first 8 h of infection, levels of mutant L2 were greatly diminished to less than 1% of input (Fig. 6C, black bars). The remarkable decrease in mutant L2 protein levels correlates well with the infectivity data (Fig. 5A) and may reflect the inability of these mutant particles to progress past the endo/lysosomal compartment. We hypothesize the mutant L2 containing virions bind and enter cells normally, trafficking to the lysosomal compartment, but the L2/genome complex fails to exit the lysosome and becomes degraded within this compartment, thereby abrogating infectivity.

Day *et al.* recently characterized the mechanisms of RG-1 neutralization, finding that the RG-1 epitope (L2 residues 17–36) is initially occluded but becomes accessible to RG-1 after a conformational change occurs on the surface of host cells [48]. Further, they demonstrate that RG-1 epitope exposure is dependent on cleavage of L2 by the cellular protease furin, a cleavage event that is essential for infection [37]. The highly conserved furin cleavage site is positioned at residues R9–R12, only ten residues upstream of the C22–C28 disulfide. Abrogation of furin cleavage through either an R9S mutation in L2 or treatment with the furin inhibitor dec.-RVKR-cmk blocks infectivity albeit the virions bind and enter cells normally, traffic and uncoat within the endosomal pathway, but fail to exit the endo/lysosomal compartment [37]. Therefore, furin-dependent display of the RG-1 epitope is likely important for endosomal escape of HPV L2/genome complexes. Our data presented herein show that in mature virions the RG-1 epitope is hidden in a buried disulfide-bonded conformation and we hypothesize that exposure of the epitope may be dependent on reduction of the C22–C28 disulfide, in addition to furin cleavage. Previous work has shown that the RG-1 antibody is reactive with L2 by western blot [39] and is therefore likely to only bind L2 17–36 in a reduced conformation, although this has not formally been examined. Our findings in the context of those of Day and colleagues [48] suggest a possible mechanistic connection between furin cleavage and reduction of L2. However, the temporal sequence of the two events is currently unclear. One possibility is that a disulfide bonded structure may be needed for furin cleavage of L2, similar to what has been shown for HIV gp160 [49], [50]. Alternatively, reduction of the C22–C28 disulfide may be a prerequisite for furin cleavage. Another possibility is that furin cleavage could precede and be required for reduction of the disulfide and exposure of the RG-1 epitope. A similar phenomenon has been shown for *Pseudomonas aeruginosa* exotoxin A (PE), a secreted ribotoxin that enters cells, traffics to the endo/lysosomal compartment and requires furin cleavage, disulfide reduction, and low pH for translocation into the cytoplasm [51], [52]. In the case of PE, furin cleavage triggers a conformational change that enables reduction of an internal disulfide by a host protein disulfide isomerase (PDI) or PDI-like enzyme, and subsequent release and cytosolic translocation of the ribotoxin domain [53]. Notably, we have observed that HPV16 infection is sensitive to bacitracin (Campos and Ozbun, unpublished results), an inhibitor of PDI activity [54]. Although PDI-family proteins are largely localized to the endoplasmic reticulum, recent reports have described a cell surface localization and function of these enzymes [55]–[57]. Furthermore, cell surface integrins and ECM components like fibronectin possess inherent PDI-like enzymatic activity [58], [59].

Ultimately the exposure of the 17–36 RG-1 epitope appears to be necessary for infection, as blocking exposure through inhibition of furin inhibition prevents exit from the endo/lysosomal compartment [37]. Interestingly an overlapping region of L2, residues 13–31, has been shown to bind to an unknown receptor on the surface of SiHa, CaSki, and HeLa cervical epithelial cells [33]. Thus it seems that the host cell receptor for the RG-1 epitope is a cell surface protein, although binding of L2 to this unknown host factor is only important for egress of genome/L2 from the endo/lysosomal compartment and not for virion uptake. The 13–31 peptide likely binds to cells in a reduced form, as these studies were performed with peptide-GFP-GST fusions purified on immobilized glutathione and eluted with *reduced* glutathione [33]. Further, 13–31 peptide binding was blocked through a valine double point mutation of residues 21 and 22 (T21V, C22V), suggesting that the free thiol group from the reduced C22 residue may directly interact with the unknown receptor. From our data we cannot ascertain whether the defect observed for the L2 cysteine mutants comes from the lack of the C22–C28 disulfide structure or whether it is a consequence of a failed interaction/binding between the exposed RG-1 epitope and an unknown host receptor due to the replacement of cysteines with serines. The high conservation of the RG-1 epitope and C22/C28 among HPV types (Fig. 1A) may be a result of selective pressures to both maintain the binding/interaction capabilities necessary for endosomal escape as well as to conceal this region from immune surveillance in the form of a disulfide bonded structure. Masking of antigenic epitopes by disulfides has been described for other viruses [60], [61]. The identification of the C22–C28 disulfide described herein warrants further experimentation to reveal the relationships between disulfide reduction, furin cleavage, exposure of the 17–36 epitope, and HPV16 infection.

## Materials and Methods

### Cell culture and infections

293TT cells (kindly provided by Chris Buck, NCI) were maintained in DMEM High Glucose (Irvine Scientific) supplemented with 10% FBS, antibiotic/antimycotic (Invitrogen), and 0.4 µg/ml Hygromycin B (Roche). HaCaT cells (a gift of N. Fusenig, DKFZ), an immortalized human keratinocyte line [44] were maintained in DMEM/Ham's F-12 medium (Irvine Scientific), supplemented with 10% FBS (Invitrogen), 4× amino acids (Invitrogen), and glutamine-penicillin-streptomycin (Invitrogen). For infections HaCaT cells were seeded in 12-well plates at 2×10^5^ cells per well. Virion stocks were vortexed and diluted to 6.67×10^7^ viral genome equivalents (vge) per ml HaCaT media. Viral inocula were added to HaCaT cells (300 µl at 100 vge per cell) and incubated at 4°C for 1 h with gentle rocking to permit viral attachment. Inocula were aspirated, cells were washed with media, and fresh media were added. Infections were allowed to proceed at 37°C for 48 h. Total RNAs were extracted from cells using TRIreagent (Invitrogen), and nucleic acid concentrations were determined by spectrophotometery. Reverse transcription (RT) of 2 µg total RNA and triplicate quantitative PCR (qPCR) reactions were performed using GeneAmp RNA PCR reagents and AmpliTaq Gold DNA polymerase (Applied Biosystems) using qPCR primers, probes, and conditions as previously described [11], [45], [46].

### Virion production, purification, and fluor-labeling

HPV16 virions were generated in 293TT cells as previously described [11], [41]. Briefly, 293TT cells, plated to ∼70% confluence in 10 cm dishes were CaPO_4_ cotransfected with 15 µg each pXULL (L1 and L2 expression plasmid) and the cloned HPV16 genome that had been excised from pBS-HPV16 and recircularized by T4 DNA ligase. Luciferase reporter transducing virions were produced by cotransfection of 293TT cells with 15 µg each of pXULL and pGL3-control (Promega). Cells were harvested 48 h post transfection by trypsinization and pelleted/resuspended in PBS+9.5 mM MgCl_2_ at 100 µl/10 cm plate. Cells were lysed by the addition of Brij58 to 0.35%. Unpackaged DNA was digested by adding exonuclease V (Epicentre plasmid-safe) to 20 U/ml and Benzonase (Sigma) to 0.3%. Lysates were then incubated overnight at 37°C for maturation [17], after which 0.17 volumes of 5 M NaCl was added and lysates were 3× freeze/thawed at −80/37°C to further break apart cellular structures. Lysates were cleared by centrifugation at 3000×g and supernatants were loaded onto discontinuous CsCl gradients made from 4 ml light (1.25 g/ml) CsCl underlaid with 4 ml heavy (1.4 g/ml) CsCl. Virions were purified by 18 h ultracentrifugation at 20,000 rpm and 4°C in a Sorvall TH-641 rotor/buckets. Viral bands were visible slightly above the gradient interface and were collected by side puncture with a 1.5” 18 gauge needle and 5 ml syringe. Virions were washed 3× and concentrated in HSB (25 mM HEPES pH 7.5, 0.5 M NaCl, 1 mM MgCl_2_) using Amicon Ultra-4 100,000 MWCO centrifugation filter units (Millipore) and stored at −80°C. Purity and L1 protein content were determined by SDS-PAGE and Coomassie Brilliant Blue staining against bovine serum albumin (BSA) standards. Virion morphology and quality was visualized by negative staining with 2% uranyl acetate and transmission electron microscopy (TEM; Hitachi 7500) at 80 kV following adsorption to a carbon-coated electron microscopy grid. Viral genome equivalent (vge) titers were determined by dot blot hybridization as previously described [45], [46]. For fluorophore labeling, ∼5 µg of HPV16 virions was diluted into 200 µl HSB and labeled with 500 ng AlexaFluor 594 (AF594) carboxylic acid succinimidyl ester (Molecular Probes) in the dark for 45 min at room temperature. Excess dye was removed by washing 3× and resuspending in 100 µl HSB using Amicon Ultra-4 100,000 kDa MWCO centrifugation filter units (Millipore). AF594-virions were stored at −80°C in HSB.

### Creation of L2 mutants

pXULL variants encoding the single C22S and C28S mutants as well as the double C22/28S mutant were generated by site directed mutagenesis using the QuikChange II XL system (Stratagene). Mutant pXULL plasmids were verified by sequence analysis and used to generate infectious virions in 293TT cells as described above.

### Sequence alignment

L2 sequences were obtained from the NCBI protein database. Accession numbers are as follows: AAD33258 (HPV16, genus alpha, species 9), AAA46955 (HPV31, genus alpha, species 9), P22165 (RhPV1, rhesus papillomavirus type 1, genus alpha, species 12), P04013 (HPV11, genus alpha, species 10), AAY86493 (HPV45, genus alpha, species 7), AAY86491 (HPV5, genus beta, species 1), NP_056743 (BPV1, bovine papillomavirus type 1, genus delta, species 4), NP 077112 (CRPV, cottontail rabbit papillomavirus, genus kappa), AAL14230 (FcPV, *Fringilla coelebs*, finch papillomavirus, genus eta). Genus and species assignments are based on recently reported phylogeny [62]. The indicated L2 residues were aligned with the MacVector software ClustalW algorithm using the BLOSUM series matrix with default settings for open gap penalty, extended gap penalty, delay divergent percentage, and protein gap parameters.

### Polyacrylamide gel electrophoresis (PAGE)

For reducing/denaturing PAGE, HPV16 virions (diluted to 8 ng/µl in HSB) were mixed with an equal volume of reducing/denaturing SDS-PAGE loading buffer (62.5 mM Tris pH 6.8, 10% glycerol, 2% SDS, 0.05% 2-mercaptoethanol, 0.05% bromophenol blue) and heated to 95°C for 5 min prior to electrophoresis on 10% polyacrylamide gels. For non-reducing/denaturing PAGE, HPV16 samples were diluted as above into HSB containing 2 mM N-ethylmaleimide (NEM, Sigma) and reacted for 1.5 h at room temperature. The purpose of the NEM reaction is to inactivate free thiols (cysteines) prior to denaturation, thereby preventing reduction of any native disulfide bonds by these free thiols during denaturation. Samples were then mixed with an equal volume of non-reducing/denaturing SDS-PAGE loading buffer (62.5 mM Tris pH 6.8, 10% glycerol, 2% SDS, 0.05% bromophenol blue) and incubated at room temperature for 10 min prior to electrophoresis on 7.5% polyacrylamide gels. Samples were transferred to PVDF membranes and L1 and L2 capsid proteins were detected by probing with 1∶10,000 dilutions of anti-[HPV16 L1] monoclonal antibody (Abcam #30908) and 1∶7500 dilutions of anti-[HPV31 L2] polyclonal antisera (DK43811, kindly provided by Richard Roden, Johns Hopkins) with 1∶25,000 dilutions of the appropriate HRP-conjugated secondary. HRP signal was detected with SuperSignal West Pico chemiluminescent substrate (Thermo Scientific).

### Thiol biotinylation assays

HPV16 virions (1 µg L1) were incubated overnight at room temperature in either 200 µl HSB (native), HSB+10 mM dithiothreitol (DTT; reduced). Samples were loaded onto Amicon Ultra-4 5,000 MWCO centrifugation filter units (Millipore) and washed 3× and resuspended in 120 µl HSB. Biotinylation reactions were then set up using 20 µl of native or DTT-reduced virion sample and 1 µg of either amine-reactive biotinyl-N-hydroxysuccinimide ester (NHS-biotin, Sigma) or thiol-reactive 1-biotinamido-4-[4′-(maleimidomethyl)cyclohexanecarboxamido]butane (BMCC-biotin, Thermo Scientific). Samples were biotinylated for 30 min at 37°C and stopped by the addition of 10 µl denaturing/reducing SDS-PAGE loading buffer (containing Tris and 2-ME to quench amine/thiol reactive reagents). Samples were denatured at 95°C for 5 min and 10 µl aliquots were electrophoresed on 7.5% polyacrylamide gels and transferred to PVDF membranes for detection of biotinylated proteins by probing with streptavidin-HRP. SDS denaturation experiments were performed by diluting HPV16 virions (500 ng L1) into 50 µl HSB+1% SDS and 1 µg BMCC-biotin. Samples were reacted for 1 h at 37°C and processed as described above.

### Mass spectrometry

Purified HPV16 virions (23 µg L1 in 40 µl HSB) were reacted with 5 mM NEM for 2 h at room temperature and mixed with 10 µl denaturing/non-reducing SDS-PAGE loading buffer, and separated on a 7.5% gel as described above. The gel was briefly stained with Biosafe Coomassie (Biorad) to visualize L1 and L2 bands. The L2 protein band (about 2 µg total L2 protein) was excised and in-gel tryptic digestion was done as previously reported [63]. The digest was done in the presence of a mass spectrometry friendly surfactant (Protease Max, Promega) to provide increased sequence coverage and the reduction and alkylation steps were excluded. Extracted peptides were dried to completion and reconstituted to 8 µl in 0.1% formic acid, 2% acetonitrile, water. Nano reversed phase HPLC was done using a 2D nanoLC (Eksigent) with buffer A consisting of 0.1% (v/v) formic acid in water and buffer B 0.1% formic acid in acetonitrile. A fused silica column self packed with duragel C18 matrix (Peeke Scientific) was used with a linear gradient from 5% B to 40% B over 60 minutes at a flow rate of 450 nl/min. The nanoHPLC was interfaced with a nanomate (Advion Biosciences) for nanoESI into the mass spectrometer. The mass spectrometer was a LCQ Deca XP Plus (Thermo Scientific) that was set in data dependent acquisition mode to perform MS/MS on the top three most intense ions with a dynamic exclusion setting of four. The DTA files were extracted from the raw data using the bioworks browser (Thermo Scientific). The data were searched against the NCBI Virus database using Mascot, as well as against the late minor capsid protein L2 (gi 4927726) sequence using SEQUEST (Thermo Scientific).

### Virion binding and entry assays

HaCaT cells were sparsely seeded on sterile glass coverslips and grown for 1.5 days. Cells were prechilled to 4°C for 15 min, media were aspirated and ∼200–300 ng AF594 labeled virus was bound to cells for 1 h at 4°C. For binding assays, unbound virus was aspirated and cells were washed once with PBS and fixed in PBS+2% paraformaldehyde for 10 min at room temperature. Cells were washed 3× with PBS and coverslips were mounted on glass slides with Vectashield® plus 4′,6-diamidino-2-phenylindole (DAPI, Vector Laboratories). Coverslips were sealed with nail polish and stored at 4°C. Entry studies were set up as described above but after AF594-virion binding cells were washed once with warm media and transferred to 37°C for 8 h. Cells were then washed 2× with PBS, fixed/permeabilized in ice-cold acetone for 10 min at −20°C, washed 3× with PBS at room temperature, and blocked overnight in PBS with 4% BSA and 0.4% goat serum. The blocking solution was aspirated and cells were incubated in mouse anti-[LAMP-1] (Abcam #25630) diluted 1∶100 in 10% blocking solution for 1 h at room temperature. Cells were washed 5× PBS and incubated in AF680-labeled goat anti-[mouse] (Molecular Probes) diluted 1∶200 to [10 ng/µl] for 1 h at room temperature. Cells were then washed 5× with PBS and mounted in Vectashield® plus DAPI (Vector Laboratories). Slides were visualized on a Zeiss LSM 510-META confocal microscope with a 63× objective. DAPI, AF594, and AF680 signals were detected with 405 nm laser diode, HeNe1 laser (543 nm), and HeNe2 laser (633 nm) excitations respectively. Images were acquired with the Zeiss LSM software and processed with Adobe Photoshop and Microsoft Powerpoint software. AF680 signal (LAMP-1 immunostaining) was pseudocolored green for contrast of co-localization with virions.

### Measurement of capsid protein levels during infection

HaCaT cells were seeded at 2×10^5^ cells/well in 12-well plates. The following day, cells were exposed to 1.25 µg purified virions in 500 µl media for 1 h at 4°C or mock infected. Cells were washed twice with media to remove unbound virus and either lysed (for 0 h time point) or replaced with fresh media and incubated at 37°C for an additional 8 h. Cell lysates were prepared by direct addition of 200 µl SDS-PAGE loading buffer to the cell monolayers. Lysates were clarified of genomic DNA by passage through QiaShredder spin columns (Qiagen) and heated to 95°C for 5 min prior to separation on a 7.5% polyacrylamide gel. Cell lysate (15 µl) was analyzed for L1 immunoblots and 30 µl of the lysate was analyzed for L2 immunodetection. Control lanes contained known amounts of HPV16 virions; 31 ng of L1 (9.5×10^8^ particles) for L1 blots and 62 ng of L1 (1.9×10^9^ particles) for L2 blots. Gels were transferred to PVDF membranes and blocked overnight at 4°C in TBST plus 2% dried milk, 2% BSA, and 1% goat serum. Blots were probed for L1 and L2 proteins as described above. Films were scanned on a BioRad GS-800 densitometer and analyzed with Quantity One software (BioRad). Mean densities were determined for the samples relative to the internal loading control band and percent capsid protein remaining after 8 h was calculated for L1 and L2.
